# Rapid and Tunable Control of Protein Stability in *Caenorhabditis elegans* Using a Small Molecule

**DOI:** 10.1371/journal.pone.0072393

**Published:** 2013-08-22

**Authors:** Ukrae Cho, Stephanie M. Zimmerman, Ling-chun Chen, Elliot Owen, Jesse V. Kim, Stuart K. Kim, Thomas J. Wandless

**Affiliations:** 1 Department of Chemical and Systems Biology, Stanford University, Stanford, California, United States of America; 2 Department of Genetics, Stanford University, Stanford, California, United States of America; 3 Department of Developmental Biology, Stanford University, Stanford, California, United States of America; Université de Montréal, Canada

## Abstract

Destabilizing domains are conditionally unstable protein domains that can be fused to a protein of interest resulting in degradation of the fusion protein in the absence of stabilizing ligand. These engineered protein domains enable rapid, reversible and dose-dependent control of protein expression levels in cultured cells and *in vivo*. To broaden the scope of this technology, we have engineered new destabilizing domains that perform well at temperatures of 20–25°C. This raises the possibility that our technology could be adapted for use at any temperature. We further show that these new destabilizing domains can be used to regulate protein concentrations in *C. elegans*. These data reinforce that DD can function in virtually any organism and temperature.

## Introduction

**Figure 1:**
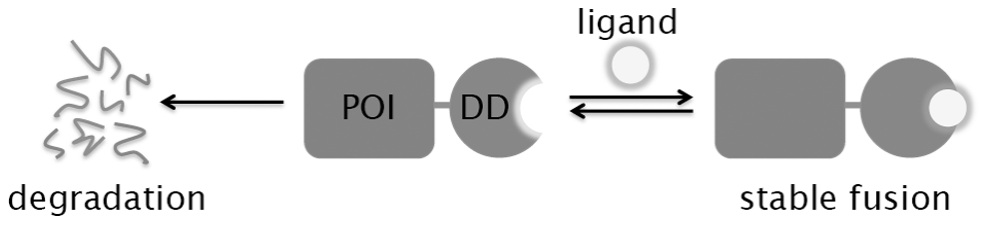
Schematic showing that the stability of a protein of interest (POI) fused to a destabilizing domain (DD) can be controlled using a high-affinity, stabilizing ligand.

One of the fundamental experimental strategies of modern molecular biology is “perturb and observe”. Investigators perturb a complex biological system and, by observing the consequences of their experimental change, infer the function of the perturbed component. Perturbations that are specific (i.e., only modulate the molecule under investigation) are desired in order for researchers to be confident in their conclusions. To this end, we developed a small, metastable protein domain whose metabolic stability relies on the presence of a high-affinity, small molecule ligand [1]. These domains, which we call destabilizing domains (DDs), can be genetically fused to any protein of interest, resulting in a fusion protein that, when expressed in eukaryotic cells, is constitutively degraded by the 26S proteasome in the absence of the stabilizing ligand. However, the addition of a cell-permeable, high-affinity ligand stabilizes the DD in a rapid, dose-dependent, and reversible manner (Figure 1).

This system has been successfully used in many different cell types [2]–[7], however the current portfolio of reagents do not reliably confer instability when expressed in cells or organisms whose optimal growth temperature is below 37°C. For example, nematodes (*C. elegans*) or zebrafish (*D. rerio*) thrive best in the temperature range of 20–25°C. Since there is an obvious need for an inducible protein expression system in both model organisms, we sought to engineer DDs that function well when expressed at temperatures closer to room temperature.

We first examined whether existing DDs, all developed at 37°C, are functional at 25°C. From this pilot experiment, we discovered that dynamic range (i.e., the difference in expression levels of a DD-fusion protein comparing the ligand-free and ligand-bound states) decreases significantly at non-optimal temperature. The stabilization by ligand binding cannot be fully achieved, and the degradation in the absence of ligand is partial. To this end, we decided to engineer new DDs based on the *E. coli* dihydrofolate reductase (ecDHFR) protein that display maximal ligand-dependent stability at or near 25°C. Herein, we report seven new ecDHFR mutants that act as DDs when fused to the N- or C-terminus of YFP.

Using one of the seven new DDs, we for the first time show that the DD system can be applied to conditionally regulate protein expression levels in *C. elegans.* Though *C. elegans* is a widely used model organism, it has relatively few options for inducible expression. At the level of DNA, previous studies have used the Cre-*LoxP* and Flp-*FRT* systems to induce gene expression by deleting repressive elements via recombinases [8]–[10]. Alternatively, a heat-shock responsive promoter can be used to drive gene expression when activated by heat shock [11]. The recently published application of the Q system to *C. elegans* provided the first example of a binary expression system capable of precise temporal and spatial control [12]. Application of the DD system to *C. elegans* would provide a genetically concise strategy for conditional regulation of gene products, and to our knowledge is the first system that provides direct, post-translational control of protein levels. To this end, we show that addition of trimethoprim, a small molecule DD ligand, can rapidly induce YFP-DD expression in *C. elegans* grown at 20°C, that degradation of YFP-DD is complete in the absence of trimethoprim, and that induced expression can be rapidly withdrawn after removal of trimethoprim.

## Results and Discussion

The temperature dependence of DDs has never been studied, so we decided to investigate how existing DDs, which were engineered in NIH 3T3 cells cultured at 37°C, perform when cultured at 25°C. We recently demonstrated that ligand-free DDs have higher propensity to unfold compared to ligand-bound DDs, thereby having a higher chance of becoming ubiquitylated and ultimately degraded by the proteasome [13]. This suggests that the ligand-dependent stabilization is primarily due to the change in protein folding state regulated by ligand binding. However, protein conformation and protein-ligand interaction may assume different energy landscapes in different temperatures, so we expected that the dynamic range of a given DD would vary according to the temperature.

**Figure 2:**
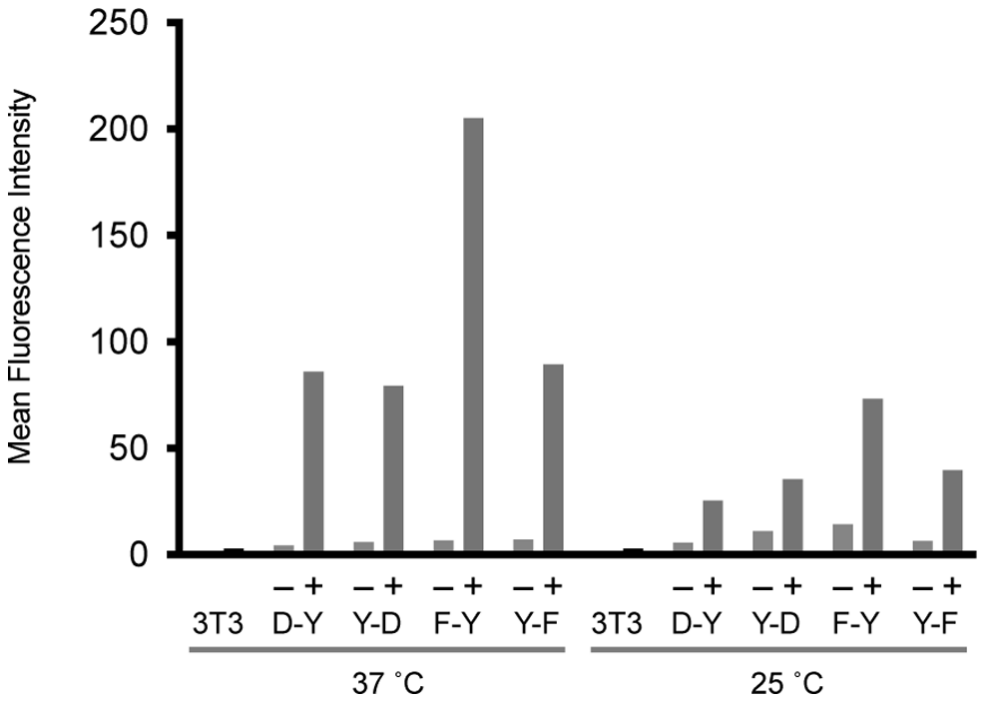
Temperature dependence of existing DDs. Destabilizing domains derived from either FKBP or ecDHFR were fused to the N-terminus of YFP (F-Y and D-Y) or the C-terminus of YFP (Y-F and Y-D). The indicated fusion proteins were stably expressed in NIH 3T3 cells for 24 h treated with vehicle (–) or stabilizing ligand (+, 2 µM Shield-1 for FKBP-derived DD and 10 µM trimethoprim for ecDHFR-derived DD) at 25°C or 37°C. The expression levels of the fusion proteins were then measured by flow cytometry. (FKBP mutations in F-Y: L106P, Y-F: E31G/R71G/K105E, DHFR mutations in D-Y: R12Y/G67S/Y100I, Y-D: R12H/N18T/A19V/G67S).

We tested two of the DD systems by preparing four constructs consisting of the FKBP-derived DD fused to the N- or C-terminus of yellow fluorescent protein (YFP) as well as the ecDHFR-derived DD fused to the N- or C-terminus of YFP. These four constructs were stably introduced into NIH 3T3 cells by retroviral transduction. After 3 days, the stabilizing ligand (Shield-1 for FKBP-derived DDs or trimethoprim for ecDHFR-derived DDs) was added to the cell culture media. Cells were then cultured at 25°C or 37°C for 24 hours before the YFP levels were analyzed by flow cytometry. As expected, the dynamic range was larger at 37°C for all four DD fusions (Figure 2). At 25°C, stabilization of the fusion protein was partial, and degradation in the absence of DD ligand was not fully achieved. Since we are seeking to broaden the scope of the DD technology, we decided to develop a new DD that will provide a large dynamic range when expressed in cells cultured at 25°C. In addition, the development of a low-temperature DD would provide evidence that DDs could be engineered for any desired experimental conditions.

For developing a low-temperature DD, we decided to use the ecDHFR protein as our template. Trimethoprim is an inexpensive small molecule with excellent pharmacological properties. Thanks to trimethoprim's superior pharmacological properties, the ecDHFR DD system has been utilized to control protein levels in rat brain [6]. To obtain ecDHFR proteins that display ligand-dependent stability, we generated a library of mutants using error-prone PCR with ecDHFR R12H G67S as a parental template. Libraries of mutants were then fused to either the N- or C-terminus of YFP, and these libraries were stably transduced into NIH 3T3 cells.

**Figure 3:**
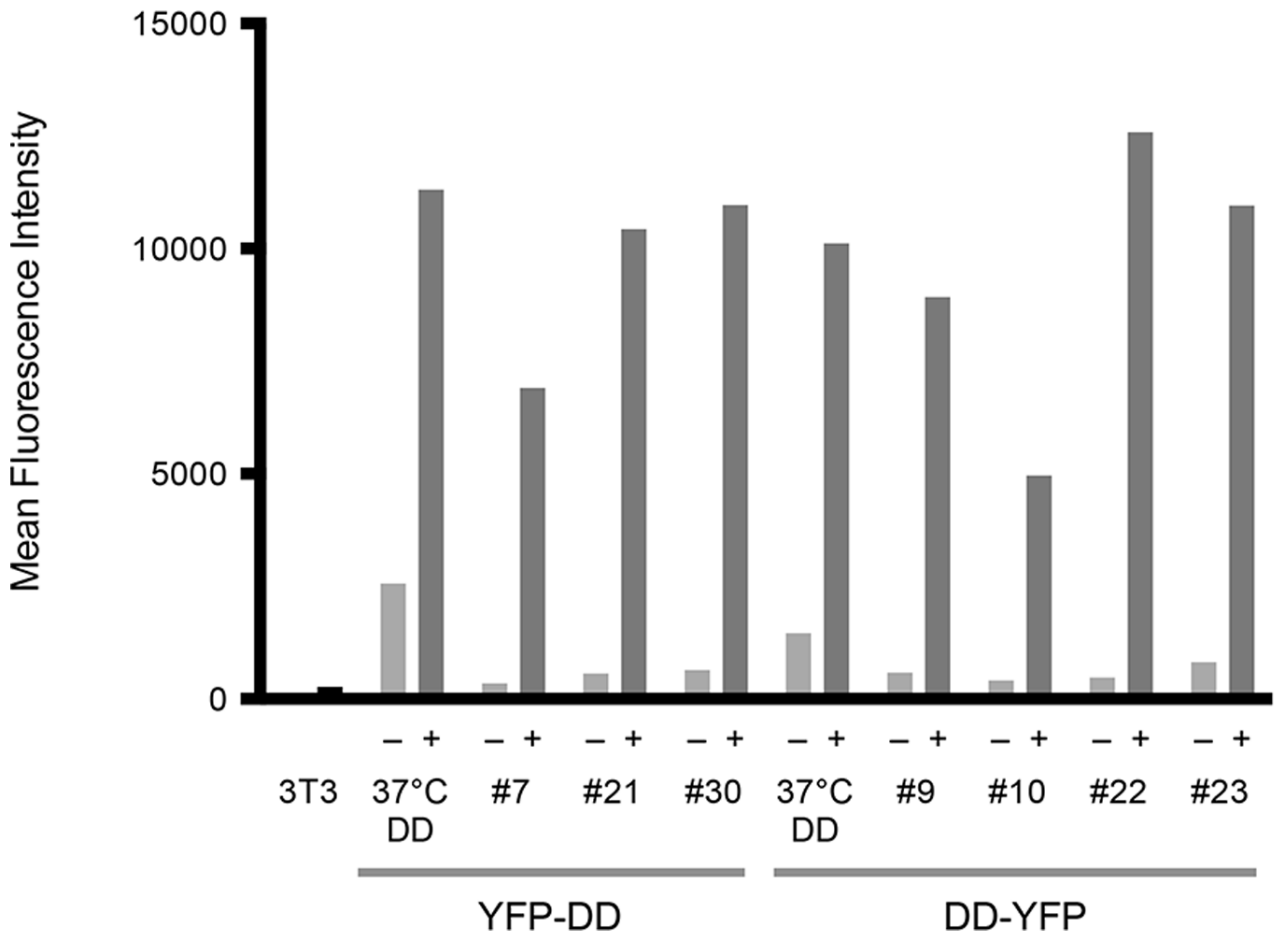
Ligand-dependent stability of new DDs screened at 25°C. NIH 3T3 cells were stably transduced with the indicated ecDHFR mutants fused to YFP and treated with vehicle (–) or 10 µM trimethoprim (+) for 24 hours at 25°C. (37°C DDs are the ones engineered at 37°C; same mutants as Y-D and D-Y in Figure 2).

Populations of NIH 3T3 cells encoding these two DD libraries underwent three rounds of fluorescence-activated cell sorting (FACS) screening. We first selected cells that highly express YFP following 24 hours of treatment with 10 µM trimethoprim at 37°C (The treatment was done at 37°C, not 25°C, for this first round because we were concerned that we would purge out too much portion of the library from the start). Next, we selected cells with low YFP levels in the absence of trimethoprim. For this second round of screening, the cells were cultured at 37°C for the most time, but 24 hours prior to FACS, they were shifted to 25°C. For the third and final round of screening, we selected cells expressing high levels of YFP following 24 hours of treatment with 10 µM trimethoprim. Specifically, the cells were cultured at 37°C prior to the addition of trimethoprim, and the cells were shifted to 25°C during the 24-hour ligand dosing period. After three rounds of FACS, genomic DNA was extracted, and genome-integrated DDs were amplified by PCR. For both the N-terminal and C-terminal libraries, 25 clones were chosen and sequenced. The candidate DDs were stably transduced back into NIH 3T3 cells, and YFP expression levels were measured in the absence and presence of 10 µM trimethoprim. Figure 3 shows clones from both libraries that display ligand-dependent stability (sequence information shown in Table 1). In the absence of trimethoprim (mock-treated with DMSO), the expression levels of YFP-fused DDs drop 25–80 fold.

**Table 1:** Sequences^a^ of ecDHFR mutants operating as DDs.

YFP – ecDHFR		ecDHFR DD – YFP	
Clone #	mutations	Clone #	mutations
7	N37D, N59D, D132G	9	N37G, P39S, K58N, G97S, H141R, N147S
21	Y100I, D132G	10	S63C, D79G, Y100H, S135P, S138N, C152R, I155T, E157G
30	Y100I	22	N23S, V78A, E120G, E134G, E154V, E157G
		23	N18D, I61T, G121V

a In addition to the mutations listed in the table, all mutants carried R12H and G67S since they were already in the parental template for mutagenesis.

Although not an absolute rule, DDs that are strongly destabilizing (i.e., very low YFP levels in the absence of ligand) also display lower YFP expression levels in the presence of the stabilizing ligand, when compared to less destabilizing mutants. DDs with this profile, such as clone #7 from the YFP-DD library and clone #10 from the DD-YFP library, may be preferred for experiments that require complete removal of a DD-fusion protein. Conversely, DDs that display greater ligand-mediated stabilization (e.g., clone #30 from the YFP-DD library or clone #23 from the DD-YFP library) may be preferred for experiments where high protein expression levels are required to elicit the phenotype under investigation. In addition, when investigators want to minimize any potential burden on the cellular protein quality control system, these clones might be more preferable.

**Figure 4:**
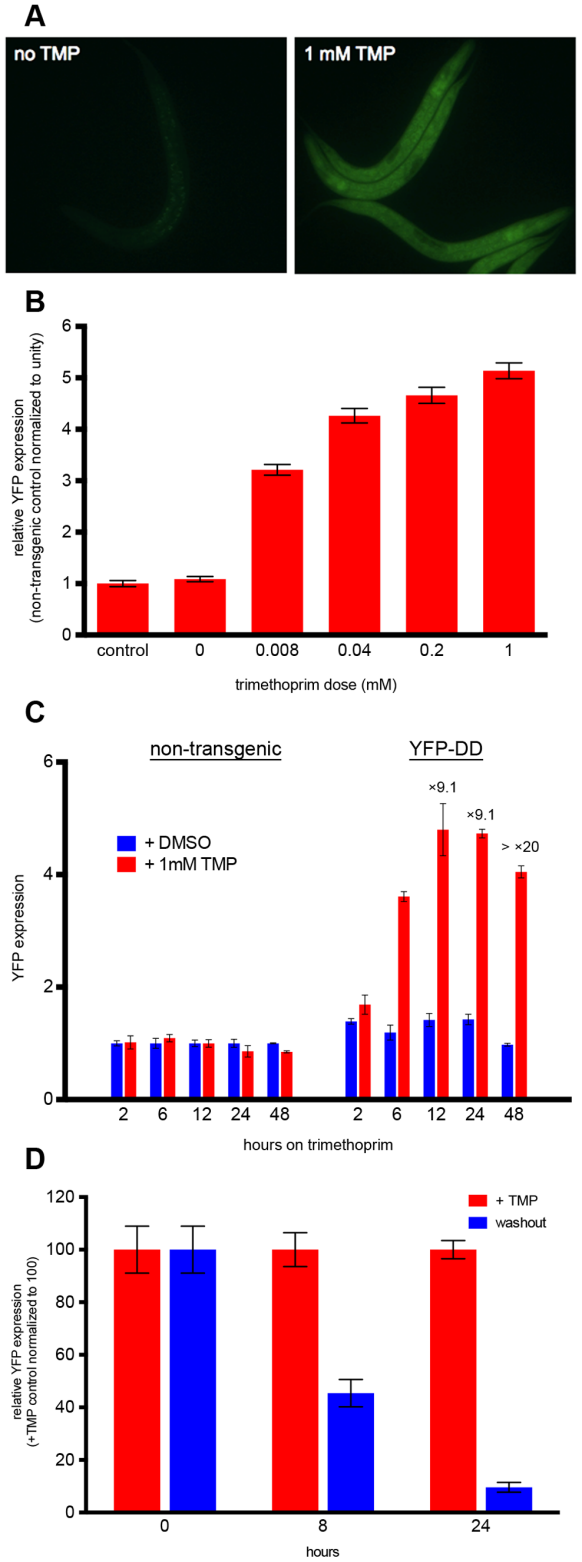
Trimethoprim rapidly stabilizes DDs expressed in *C.* *elegans* in a dose-dependent manner. (A) Representative images of transgenic worms expressing *eft-3pro*:YFP-DD clone #7 from a single copy MosSci insertion grown in the absence or presence of 1 mM trimethoprim (TMP) for 24 hours at 20°C. (B) Wild type (N2) and YFP-DD transgenic worms were placed on plates containing 0.008, 0.04, 0.2, or 1 mM trimethoprim or control plates (DMSO vehicle only) as synchronized L1 larvae and imaged after 24 hours at 20°C. Graph shows average fold-induction of YFP expression relative to a non-transgenic control at increasing doses of trimethoprim (n>20 worms per dose). Error bars are ± SEM. (C) Wild-type (N2) or YFP-DD transgenic worms were placed on plates containing either 1 mM trimethoprim or DMSO vehicle control and imaged after 2, 6, 12, 24, and 48 h at 20°C. The graph shows the YFP expression of trimethoprim treated worms normalized to age-matched DMSO treated worms at each time point (n>20 worms per dose). Error bars are ± SEM. (D) YFP-DD transgenic worms were placed on plates containing 1 mM trimethoprim or DMSO vehicle control as L1s and grown for 24 hours, at which point one group was maintained on trimethoprim (“+TMP”), one was moved from trimethoprim to control plates (“washout”), and one was maintained on DMSO control plates. The graph shows background subtracted YFP expression of washout worms relative to +TMP worms at each timepoint (n>20 worms per condition). Error bars are ± SEM.

Encouraged, we sought to apply these new DDs in *C. elegans*, which is a widely used model organism that is maintained in the laboratory at 15–25°C**.** We chose to focus on clone #7 from the YFP-DD library, because this new DD showed the largest dynamic range between vehicle-treated and trimethoprim-treated cells of the seven mutants shown in Figure 3. We generated worms expressing YFP-DD clone #7 under the control of the ubiquitous *eft-3* promoter by single copy MosSci integration into the ttTi4348 locus on chromosome I. Synchronized L1 larvae were grown on NGM plates containing 0.008, 0.04, 0.2, or 1 mM trimethoprim dissolved in DMSO or control plates (DMSO vehicle) for 24 hours at 20°C. In the absence of trimethoprim, there was no detectable YFP expression in DD-YFP worms compared to N2 non-transgenic controls, indicating complete degradation of YFP-DD. With increasing doses of trimethoprim, YFP expression levels increased, with a minimum of 30-fold dynamic range at 8 µM trimethoprim and a maximum of 45-fold at 1 mM trimethoprim (Fig. 4A−B). At the 1 mM dose, trimethoprim had no effect on larval development rate (Fig. S1).

Next, we examined the kinetics of gene induction upon the addition of trimethoprim. Synchronized L1 larvae were grown on NGM plates containing 1 mM trimethoprim or DMSO control plates for 2, 6, 12, 24, and 48 hours. YFP expression was partially induced after 6 hours and increased with time, reaching a maximum of greater than 20-fold induction after 48 hours. Trimethoprim did not induce autofluorescence in non-transgenic controls at any timepoint (Fig. 4C). Finally, we tested whether induced YFP-DD expression could be reversed through withdrawal of trimethoprim, providing a tool for loss-of-function as well as gain-of-function experiments. Synchronized L1 larvae were grown on control plates or plates containing 1 mM trimethoprim for 24 hours, and then either maintained on trimethoprim as a positive control, maintained on control plates as a negative control, or transferred from trimethoprim to control plates (washout experiment). After 8 hours of washout, YFP expression was less than half that of worms that had been continually maintained on trimethoprim, and by 24 hours had reached nearly the level of control animals (Fig. 4d). These results indicate that the DD system can be successfully used to rapidly induce and withdraw gene expression in *C. elegans*, with little to no background expression in the absence of the stabilizing ligand and robust and tunable expression by addition of the ligand.

In conclusion, we have developed a DD that can be utilized in *C. elegans*. We showed that a DD can be optimized for use at temperatures other than 37°C, and that this new low-temperature DD gives full control of protein stability in *C. elegans*. To the best of our knowledge, there has not been a system for *C. elegans* where a protein concentration can be controlled directly at the protein level. With this toolkit in hand, *C. elegans* researchers will now be able to perform not only gain- and loss-of-function experiments but also do dose titration of a protein in cells.

## Materials and Methods

### ecDHFR Library & Retroviral Gene Expression

The ecDHFR mutant library was prepared using error-prone PCR and nucleotide analog (8-oxo-dGTP) mutagenesis. The ecDHFR R12H G67S mutant was used as a parental template. The estimated size of the library was 3×10^4^ for the DD-YFP library and 1.5×10^5^ for the YFP-DD library [6]. These fusion genes were cloned into pBMN retroviral expression vectors. Phoenix ecotropic packaging cells (φNX-E) were transfected with the respective libraries using Lipofectamine 2000 in Opti-MEM (2∶5 ratio, µg DNA: µL Lipofectamine). Then, NIH 3T3 cells in complete media (DMEM supplemented with 10% FBS, 100 U/mL penicillin, and 100 µg/mL streptomycin) were added the filtered retrovirus solution supplemented with polybrene (4 µg/mL). After 4 h the retroviral media was replaced with complete media to avoid multiple infections, and cells were cultured for 48–72 h to allow for viral integration.

### Cell Culture & Flow Cytometry

Unless stated otherwise, cells were cultured at 37°C. For ligand dosing, stably transduced NIH 3T3 cells were incubated with Shield-1 (2 µM), trimethoprim (10 µM), or DMSO for 24 h at 25°C or 37°C. For flow cytometry cells were prepared as a suspension (in growth media) using trypsin, then kept on ice, and analyzed at the Stanford Shared FACS Facility.

### Expression of YFP-DD in *C. elegans*

All *C. elegans* strains were handled and maintained as described previously [14]. Transgenic YFP-DD strains were constructed by MosSci insertion into the ttTi4348 locus on chromosome I in an *unc-119(ed3)* background as described by Frøkjær-Jensen et al [15]. Briefly, YFP-DD clone #7 was cloned after the ATG of the strong and ubiquitous *eft-3* promoter and inserted into the pCFJ352 vector containing ttTi4348 mos sites and the *C. briggsae unc-119* gene. The resulting plasmid was microinjected into *unc-119(ed3)* insertion strain EG6701 with the Mos1 transposase (pCFJ601), the negative selection marker *Phsp:peel-1* (pMA122), and 2 mCherry co-injection markers. *unc-119(+)* rescue lines were screened for insertion as described in [15] for survival of heat shock, loss of mCherry co-injection markers, and by PCR across the junction of the *unc-119* gene and chromosome I. A single positive insertion was homozygosed to generate strain SD1913 and used for all subsequent experiments.

Trimethoprim plates were prepared by adding an appropriate amount of a stock solution of 100 mM Trimethoprim dissolved in DMSO to standard NGM media after autoclaving to produce plates with a final concentration of 0.008, 0.04, 0.2, or 1 mM trimethoprim. Plates containing the same concentration of DMSO alone as in the 1 mM Trimethoprim plates were used as a control. YFP-DD (clone #7) and N2 worms were synchronized using hypochlorite to extract eggs and then hatched overnight in M9 to generate arrested L1 stage larvae. L1 larvae were placed on plates containing one of four doses of trimethoprim or DMSO control plates that had been seeded with 10× concentrated overnight cultures of *E. coli* strain OP50. After 24 h, worms were imaged on a fluorescent microscope and fluorescent intensity was measured using ImageJ [16]. n>20 worms for each condition.

For the kinetics experiment, synchronized arrested DD-YFP or wild-type (N2) L1 stage larvae were placed on 1 mM trimethoprim plates or control plates containing an equal concentration of DMSO (prepared as above). Both trimethoprim-treated and control worms were imaged at 2, 6, 12, 24, and 48 h and fold-induction was calculated according to the following formula: [YFP-DD^TMP, × hour^ – non-transgenic^TMP, × hour^]/[YFP-DD^DMSO, × hour^ – non-transgenic^DMSO, × hour^]. n>20 worms for each condition.

For the withdrawal experiment, synchronized L1 larvae were grown on 1 mM trimethoprim plates or DMSO control plates for 24 hours, and then either maintained on trimethprim plates, maintained on control plates, or moved from trimethoprim to control plates. Worms were imaged at 0, 8, and 24 hours after the start of the washout period. Fold change was calculated using the same formula above.

For the development time experiment (Fig. S1), synchronized starved L1 larvae were grown on standard NGM plates, NGM plates with 1% DMSO (v/v), or 1 mM trimethoprim dissolved in 1% DMSO for 50 hours at 20°C. The percent of worms at each developmental stage (L1, L2, L3, L4, and young adult) was assessed visually. Two replicates were performed for each condition. n>80 worms per replicate in all conditions.

## Supporting Information

Figure S1 **Trimethoprim has no effect on** ***C. elegans*** **development rate.** Synchronized starved L1 animals were put on standard NGM plates, NGM plates with DMSO (1% v/v), and NGM plates with TMP dissolved in DMSO (1% v/v DMSO, 1 mM TMP). The graph shows the percentage of each stage after 50 hours (n >80 for each bar). Trimethoprim and DMSO did not affect development rate (YA: young adult; L1-4: four larval stages). Each condition was run in duplicate.
(DOCX)
