# Supplementary material for: Rapid and Tunable Control of Protein Stability in Caenorhabditis elegans Using a Small Molecule
Source: PLoS One. 2013 Aug 22;8(8):e72393. doi: 10.1371/journal.pone.0072393 (PMC3750007; doi:10.1371/journal.pone.0072393)
Supplement: Figure S1 — Trimethoprim has no effect on C. elegans development rate. Synchronized starved L1 animals were put on standard NGM plates, NGM plates with DMSO (1% v/v), and NGM plates with TMP dissolved in DMSO (1% v/v DMSO, 1 mM TMP). The graph shows the percentage of each stage after 50 hours (n >80 for each bar). Trimethoprim and DMSO did not affect development rate (YA: young adult; L1-4: four larval stages). Each condition was run in duplicate. (DOCX) [file pone.0072393.s001.docx]

**Rapid and Tunable Control of Protein Stability
in *Caenorhabditis elegans* Using a Small Molecule**

Ukrae Cho^1^, Stephanie M. Zimmerman^2^, Ling-chun Chen^1^,
Elliot Owen^2,3^, Jesse V. Kim^2,3^, Stuart K. Kim^2,3^ and Thomas J. Wandless^1^*

Departments of ^1^Chemical and Systems Biology, ^2^Genetics, and ^3^Developmental Biology
Stanford University, Stanford, CA, USA

* Email: wandless@stanford.edu

**Figure S1**


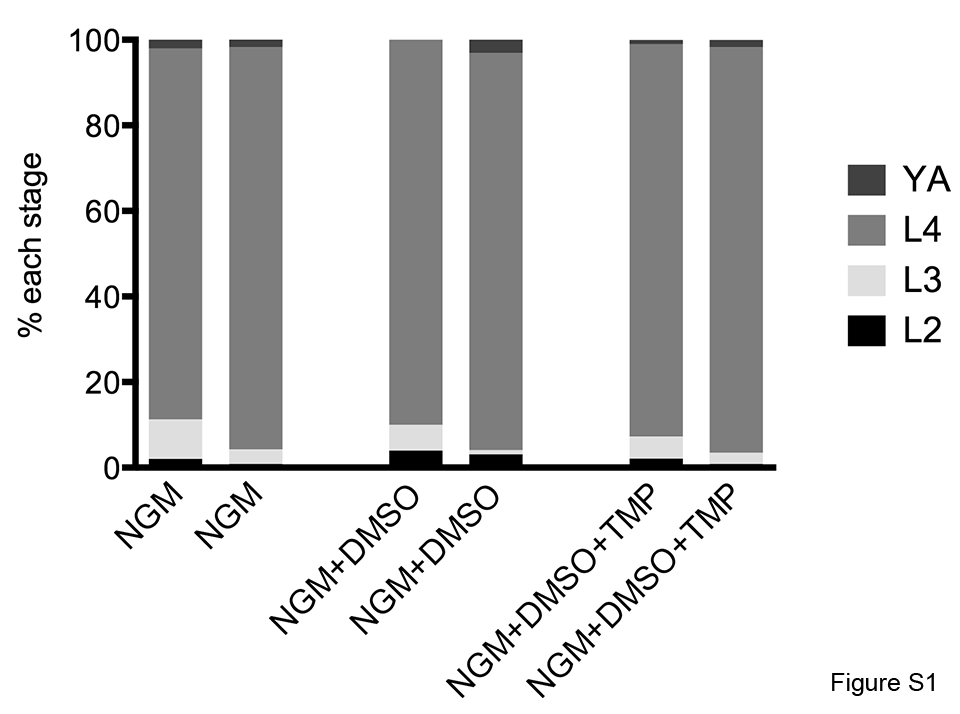


**Figure S1. Trimethoprim has no effect on *C. elegans* development rate.** Synchronized starved L1 animals were put on standard NGM plates, NGM plates with DMSO (1% v/v), and NGM plates with TMP dissolved in DMSO (1% v/v DMSO, 1 mM TMP). The graph shows the percentage of each stage after 50 hours (n > 80 for each bar). Trimethoprim and DMSO did not affect development rate (YA: young adult; L1-4: four larval stages). Each condition was run in duplicate.
